# Monocyte Dysfunction Detected by the Designed Ankyrin Repeat Protein F7 Predicts Mortality in Patients Receiving Veno-Arterial Extracorporeal Membrane Oxygenation

**DOI:** 10.3389/fcvm.2021.689218

**Published:** 2021-07-19

**Authors:** Patrick M. Siegel, Lukas Orlean, István Bojti, Klaus Kaier, Thilo Witsch, Jennifer S. Esser, Georg Trummer, Martin Moser, Karlheinz Peter, Christoph Bode, Philipp Diehl

**Affiliations:** ^1^Department of Cardiology and Angiology I, University Heart Center Freiburg - Bad Krozingen, Faculty of Medicine, University of Freiburg, Freiburg, Germany; ^2^Institute of Medical Biometry and Statistics, Faculty of Medicine and Medical Center, University of Freiburg, Breisgau, Germany; ^3^Department of Cardiovascular Surgery, University Heart Center Freiburg - Bad Krozingen, Faculty of Medicine, University of Freiburg, Freiburg, Germany; ^4^Atherothrombosis and Vascular Biology Laboratory, Baker Heart and Diabetes Institute, Melbourne, VIC, Australia; ^5^Department of Medicine, Central Clinical School, Monash University, Melbourne, VIC, Australia; ^6^Baker Department of Cardiometabolic Health, University of Melbourne, Melbourne, VIC, Australia

**Keywords:** extracorporeal membrane oxygenation, monocyte, Mac-1, inflammation, DARPin^®^, activation

## Abstract

**Background:** Veno-arterial extracorporeal membrane oxygenation (VA-ECMO) is used for critically ill patients requiring hemodynamic support but has been shown to induce an inflammatory response syndrome potentially leading to severe complications and poor outcome. Monocytes are comprised of different subsets and play a central role in the innate immune system. The *unique* small binding proteins, Designed Ankyrin Repeat Protein “F7” and single chain variable fragment “MAN-1,” specifically detect the activated conformation of the leukocyte integrin Mac-1 enabling the highly sensitive detection of monocyte activation status. The aim of this study was to characterize monocyte function and heterogeneity and their association with outcome in VA-ECMO patients.

**Methods:** VA-ECMO patients were recruited from the ICUs of the University Hospital in Freiburg, Germany. Blood was sampled on day 0 and day 3 after VA-ECMO placement, after VA-ECMO explantation and from healthy controls. Monocyte subset distribution, baseline activation and stimulability were analyzed by flow cytometry using the unique small binding proteins F7 and MAN-1 and the conventional activation markers CD163, CD86, CD69, and CX3CR1. Furthermore, expression of monocyte activation markers in survivors and non-survivors on day 0 was compared. Simple logistic regression was conducted to determine the association of monocyte activation markers with mortality.

**Results:** Twenty two patients on VA-ECMO and 15 healthy controls were recruited. Eleven patients survived until discharge from the ICU. Compared to controls, baseline monocyte activation was significantly increased, whereas stimulability was decreased. The percentage of classical monocytes increased after explantation, while the percentage of intermediate monocytes decreased. Total, classical, and intermediate monocyte counts were significantly elevated compared to controls. On day 0, baseline binding of F7 was significantly lower in non-survivors than survivors. The area under the ROC curve associated with mortality on day 0 was 0.802 (*p* = 0.02).

**Conclusions:** Distribution of monocyte subsets changes during VA-ECMO and absolute classical and intermediate monocyte counts are significantly elevated compared to controls. Monocytes from VA-ECMO patients showed signs of dysfunction. Monocyte dysfunction, as determined by the *unique tool* F7, could be valuable for predicting mortality in patients receiving VA-ECMO and may be used as a novel biomarker guiding early clinical decision making in the future.

## Introduction

Veno-arterial extracorporeal membrane oxygenation (VA-ECMO) is being increasingly used as a last resort for critically ill patients with circulatory failure. Although the underlying technology has substantially improved, the mortality rate in these patients remains high at over 50% (1, 2). Contributing to this high mortality rate are severe, life-threatening complications such as bleeding, thrombosis, and capillary leakage syndrome which have been associated with a systemic inflammatory response syndrome occurring after ECMO initiation. This overshooting inflammatory response is triggered by contact of blood components with extracorporeal surfaces (3).

The inflammatory reaction to ECMO therapy is complex. Recent studies report altered function and response to stimulation of immune cells in ECMO patients, also referred to as *immunoparalysis*. It has been suggested that this phenomenon might also affect outcome in these patients, e.g., by increased vulnerability toward infection (4, 5).

Monocytes play a crucial role in the development and progression of a wide range of inflammatory conditions (6–8). They can be differentiated according to their expression of the surface markers CD14 and CD16 into classical (CD14^++^CD16^−^), intermediate (CD14^++^CD16^+^), and non-classical (CD14^+^CD16^++^) monocytes, each with distinct functions (9). Only limited information is available on monocyte subset *distribution* and *function* in ECMO patients.

Several markers of monocyte activation have been described including CD163 (10, 11), CD86 (12, 13), CD69 (14, 15), CX3CR1 (16), and the leukocyte integrin Mac-1 [=CD11b or α_M_β_2_-integrin (17)]. Importantly, Mac-1 exists in a resting and activated conformation on the monocyte surface depending on the activation status and the change in integrin conformation occurs within minutes of leukocyte activation (18, 19). The sensitivity of detecting monocyte activation can be dramatically increased by specifically detecting the activated conformation of Mac-1 using the following *unique tools:* the small binding proteins, single chain variable fragment (scFv) MAN-1 and Designed Ankyrin Repeat Protein (DARPin) F7.

These small binding proteins, compared to conventional immunoglobulins, are more stable, have similar or even higher affinities to their targets and are more easily produced and selected, entailing lower costs (20). MAN-1 and F7 have previously been used to detect monocyte activation in a clinical setting, e.g., in septic patients or patients with myocardial infarction (19, 21).

The aim of this study was to characterize monocyte function and heterogeneity and their association with outcome in VA-ECMO patients.

## Materials and Methods

### Patient Recruitment

Patients were recruited prospectively from the intensive care units of the medical and heart surgical intensive care wards of the University Hospital in Freiburg, Germany from December 2019 until December 2020. Daily screening of the patient data management system was performed to identify patients receiving VA-ECMO. Patients were eligible if they were 18 years or older, had no hematological malignancies and a hemoglobin value above 8 g/dl. Blood was carefully drawn from an arterial line from patients after VA-ECMO implantation on day 0 (“d0,” <24 h after VA-ECMO initiation), day 3 (“d3,” 72 h ± 12 h after VA-ECMO initiation) and after explantation (“post explant.,” <12 h after VA-ECMO explantation). Blood was immediately transferred to the laboratory where flow cytometric analysis was carried out. Clinical and laboratory parameters were obtained from the electronic patient data management system. Healthy volunteers were free of disease and had not taken any drugs in the past 14 days and blood was taken by antecubital vein puncture.

### Indications, Implantation, and Management of VA-ECMO

The decision on the placement of VA-ECMO was made by an experienced ECMO physician. Implantation and management were carried out as described previously (22). Most commonly, venous cannulas had a diameter of 21-23 F and arterial cannulas were 15-17 F. Distal limb perfusion was added if necessary.

Patients on VA-ECMO regularly received transfusions as indicated to maintain hemoglobin levels above 8 g/dl and a platelet count above 50,000/μl.

VA-ECMO was carried out using the Stöckert^®^ centrifugal pump (LivaNova PLC, London, United Kingdom), the Maquet Cardiohelp Systems with an HLS Set Advanced (Maquet Cardiopulmonary GmbH, Rastatt, Germany), the CARL system (Resuscitec, Freiburg, Germany) or the Deltastream system (Xenios AG, Heilbronn, Germany). All systems included one oxygenator.

### Flow Cytometry

#### Antibodies

All antibodies were ordered from Biolegend, USA. 8.5 μl of the following antibodies were added to the Master-Mix: PerCP-Cy5.5 anti-HLA-DR (clone L243), PE anti-CD14 (clone HCD14), Pacific Blue anti-CD16 (clone 3G8), APC anti-CD45 (clone HI30), PE/Cy7 anti-CD66b (clone G10F5) and the master-mix was stored on ice in the dark after dilution with 45 μl PBS. 5 μl of Master-Mix was added per sample. The following FITC-conjugated antibodies were used to detect monocyte activation: anti-CX3CR1 (1:10 dilution, clone 2A9-1), anti-CD69 (1:5 dilution, clone FN50), anti-CD86 (1:10 dilution, clone BU63), anti-CD163 (1:10 dilution, clone GHI/61), and 5 μl were added to the specified sample. Adequate isotype controls were prepared. DARPin F7 was produced and purified as described previously (21). MAN-1 was kindly provided by Prof. Karlheinz Peter, Melbourne, Australia. DARPin F7 (F_c_ = 2.5 μg/ml) and MAN-1 (F_c_ = 10 μg/ml) are unconjugated but feature a His-tag which allows detection by an Alexa Fluor 488 anti-His-tag antibody (1 μl per sample).

#### Protocol and Staining Procedure

100 μl of citrated blood was added per tube followed by stimulation with phorbol 12-myristate 13-acetate (PMA, 200 nM, or PBS for unstimulated samples, 15 min, 37°C). After red blood cell lysis (BD FACS Lysing Solution, BD, USA; 20 min, on ice), samples were washed with 2 ml of PBS + Ca^2+^/Mg^2+^ and resuspended in 100 μl of PBS + Ca^2+^/Mg^2+^. DARPin F7, MAN-1, anti-CD163, anti-CD69, anti-CD86 and anti-CX3CR1, and the Mastermix were added (15 min, on ice), followed by addition of the secondary antibody (anti-His-Tag Alexa, 15 min on ice). 400 μl of diluted Cell FIX^TM^ solution (BD, USA) were added and samples were read on a BD FACS Canto II Flow Cytometer at medium flow rate.

Binding of MAN-1, F7, anti-CD163, anti-C86, anti-CD69, and anti-CX3CR1 was recorded as percentage after gating for classical monocytes using adequate isotype controls as previously described (21). Marker expression on monocytes was assessed in unstimulated samples (=*baseline*), and PMA-stimulated samples (=*stimulability*). In brief, the positive population was defined by a gate including the top 1% of the population in the FITC isotype control sample (anti-CD163, anti-C86, anti-CD69, and anti-CX3CR1) or the top 1% in the secondary Alexa-Fluor 488 anti-His-tag antibody only sample (F7 & MAN-1). The population shifting into this gate in unstimulated or stimulated samples was recorded as percentage. HLA-DR expression on classical monocytes was recorded as PerCP-Cy5.5 mean fluorescence intensity.

Monocyte subsets were defined by their expression of CD14 and CD16. Classical (CD14^++^CD16^−^), intermediate (CD14^++^CD16^+^), and non-classical (CD14^+^CD16^++^) monocytes were identified in the following way: after excluding CD66b^+^ events cells, monocytes were pre-gated according to their location in the FSC/APC CD45 plot. Monocytes were then identified by their expression of HLA-DR and CD14. HLA-DR^+^CD14^+^ cells were then displayed in a PE CD14/Pacific Blue CD16 gate and subpopulations were identified as described previously using adequate isotype controls (23).

An extra sample was transferred to a Trucount^TM^ tube (BD, USA) to allow absolute quantification of cells. 10,000 Trucount^TM^ beads were recorded and monocyte concentration per ml was calculated as described in the manufacturer's instructions. Total monocyte count per ml for each patient was calculated by summarizing the number of classical, intermediate and non-classical monocytes. In the other samples 5,000 monocytes were recorded in the HLA-DR/CD14 gate. Data were analyzed using FlowJo V10.6.0.

### Statistics

Variables are presented as mean ± SEM or median (interquartile range). To account for repeated measures per patient, mixed effects models, which allow missing data, were used to analyze differences between means across different time points. Unpaired *t*-tests were used to analyze differences of means at single time points. Simple logistic regression analysis was performed to determine association of monocyte markers on day 0 with mortality. Areas under the receiver operating characteristics (ROC) curve were calculated to determine predictive accuracy of these parameters. A *p*-value ≤ 0.05 was considered statistically significant. Analysis was performed using GraphPad Prism V9.0 (GraphPad Software, San Diego, California, USA).

## Results

In a first step, the ability to detect monocyte activation in response to PMA stimulation was validated for all monocyte surface markers (F7, MAN-1, CX3CR1, CD163, CD86, CD69) in a group of healthy volunteers. These healthy volunteers also later served as a control group since monocyte function was assumed to be unaltered by medication or disease allowing them to serve as a reference. Fifteen healthy controls were recruited with a median age of 26 years (23–31). Seven were female, 8 were male. We found a significant increase in binding for all surface markers in response to stimulation with PMA. Activation-specific binding was particularly pronounced for the *unique tools* F7 and MAN-1 (Supplementary Figure 1).

Twenty-two patients receiving veno-arterial ECMO were recruited from December 2019 to December 2020. Eleven patients survived until discharge from the intensive care wards and were considered as “survivors.” Eight patients were females. Thirteen patients were recruited from the medical intensive care wards, nine patients were recruited from the heart surgical intensive care ward. Seven patients received ECMO during cardiopulmonary resuscitation (eCPR) and 15 patients due to severe cardiogenic shock. The median sequential organ failure assessment (SOFA) score (Q1-Q3) was 11 (9–13). Blood was obtained from all patients on day 0. We were able to take blood from 13 patients on day 3 and 13 patients after ECMO explantation. The reduced number of patients on day 3 was due to early weaning off ECMO (two patients) or early death (seven patients). Patient characteristics and laboratory parameters are presented in Table 1.

**Table 1:** Clinical characteristics, laboratory parameters, and ventilation settings of VA-ECMO patients on day 0.

**Parameter**	**VA-ECMO patients**
Patients, *n* (%)	22 (100)
Age, y (Q1-Q3)	63 (52-73)
Survivors, *n* (%)	11 (50)
Female, *n* (%)	8 (37)
**Type of VA-ECMO**, ***n*** **(%)**	
Stöckert Sorin Maquet Deltastream CARL	12 (55) 7 (32) 2 (9) 1 (4)
Days on ECMO (median, Q1-Q3)	6.0 (3.8-7.0)
ECMO Blood Flow (l/min, Q1-Q3)	4.3 (3.8-4.8)
**Indication for VA-ECMO**, ***n*** **(%)**	
Cardiogenic shock (due to)	15 (68)
Postoperative/postinterventional	7 (32)
Myocardial infarction	2 (9)
Ischemic cardiomyopathy	1 (4)
Unknown cardiomyopathy	1 (4)
Endocarditis	1 (4)
Pulmonary embolism	1 (4)
After CPR	1 (4)
Mitral regurgitation	1 (4)
eCPR (due to)	1 (4)
Myocardial ischemia	1 (4)
Unknown	1 (4)
Cardiovascular disease, *n* (%)	15 (68)
Atrial fibrillation, *n* (%)	8 (36)
Diabetes mellitus, *n* (%)	2 (9)
Hypertension, *n* (%)	7 (32)
Active smoker, *n* (%)	3 (14)
Hypercholesterolemia, *n* (%)	4 (18)
Cancer, *n* (%)	0 (0)
Acute renal failure, *n* (%)	15 (68)
Continuous hemodialysis, *n* (%)	9 (41)
Heparin, *n* (%)	21 (95)
Dual anti-platelet therapy, *n* (%)	9 (41)
Immunosuppression, *n* (%)	1 (4)
Received transfusions, *n* (%)	22 (100)
Cytosorb, *n* (%)	1 (4)
Mechanical ventilation, *n* (%)	22 (100)
SOFA score (Q1-Q3)	11.0 (9.0-13.0)
WBC (×10^3^ /μl, Q1-Q3)	9.6 (7.5-14.0)
Platelets (×10^3^ /μl, Q1-Q3)	102.0 (74.0-140.0)
Hb (g/dl, Q1-Q3)	8.7 (8.3-9.4)
Creatinine (mg/dl, Q1-Q3)	1.7 (1.0-2.5)
Urea (mg/dl, Q1-Q3)	58.0 (33.0-77.0)
Bilirubin (mg/dl, Q1-Q3)	2.4 (1.5-3.3)
AST (U/l, Q1-Q3)	161.0 (70.3-437.5)
ALT (U/l, Q1-Q3)	63 (25.3-123.8)
CRP (mg/l, Q1-Q3)	54.1 (25.3-101.0)
IL-6 (pg/ml)	378.0 (297.5-1007)
Ferritin (ng/ml, Q1-Q3)	371 (203.0-4163)
Lactate (mmol/l, Q1-Q3)	3.25 (1.4-5.9)
p_a_O_2_ (mmHg, Q1-Q3)	111 (76.8-167.3)
p_a_CO_2_ (mmHg, Q1-Q3)	39.4 (35.3-45.5)
F_i_O (%, Q1-Q3)	50.0 (40.0-50.0)
PEEP (mbar, Q1-Q3)	8.0 (7.0-10.0)
Respiratory rate (/min, Q1-Q3)	14.0 (12.0-18.0)

*Data are presented as median (interquartile range, Q1-Q3) or number of patients (%). Denominator of the percentage is the total number of subjects in the group. Parameters from the patient data management system that were closest to the time point of blood sampling for flow cytometric analysis are presented. ALT, alanine aminotransferase; AST, aspartate aminotransferase; CRP, C-reactive protein; eCPR, extracorporeal cardiopulmonary resuscitation; F_i_O, fraction of inspired oxygen; PEEP, positive end-expiratory pressure; SOFA, sequential organ failure assessment score; WBC, white blood cells*.

We then investigated expression of activation-specific parameters on monocytes from VA-ECMO patients on day 0, day 3, and after explantation. We show the expression of these parameters on unstimulated (=*baseline*) and PMA-stimulated monocytes (=*stimulability*) and compare these parameters to our healthy control group.

According to most parameters, baseline monocyte activation and monocyte stimulability did not change significantly while patients were on ECMO and even after explantation. However, there were significant differences in baseline monocyte activation and stimulability compared to healthy controls which we will describe in detail for the different parameters investigated.

**Figure 1:**
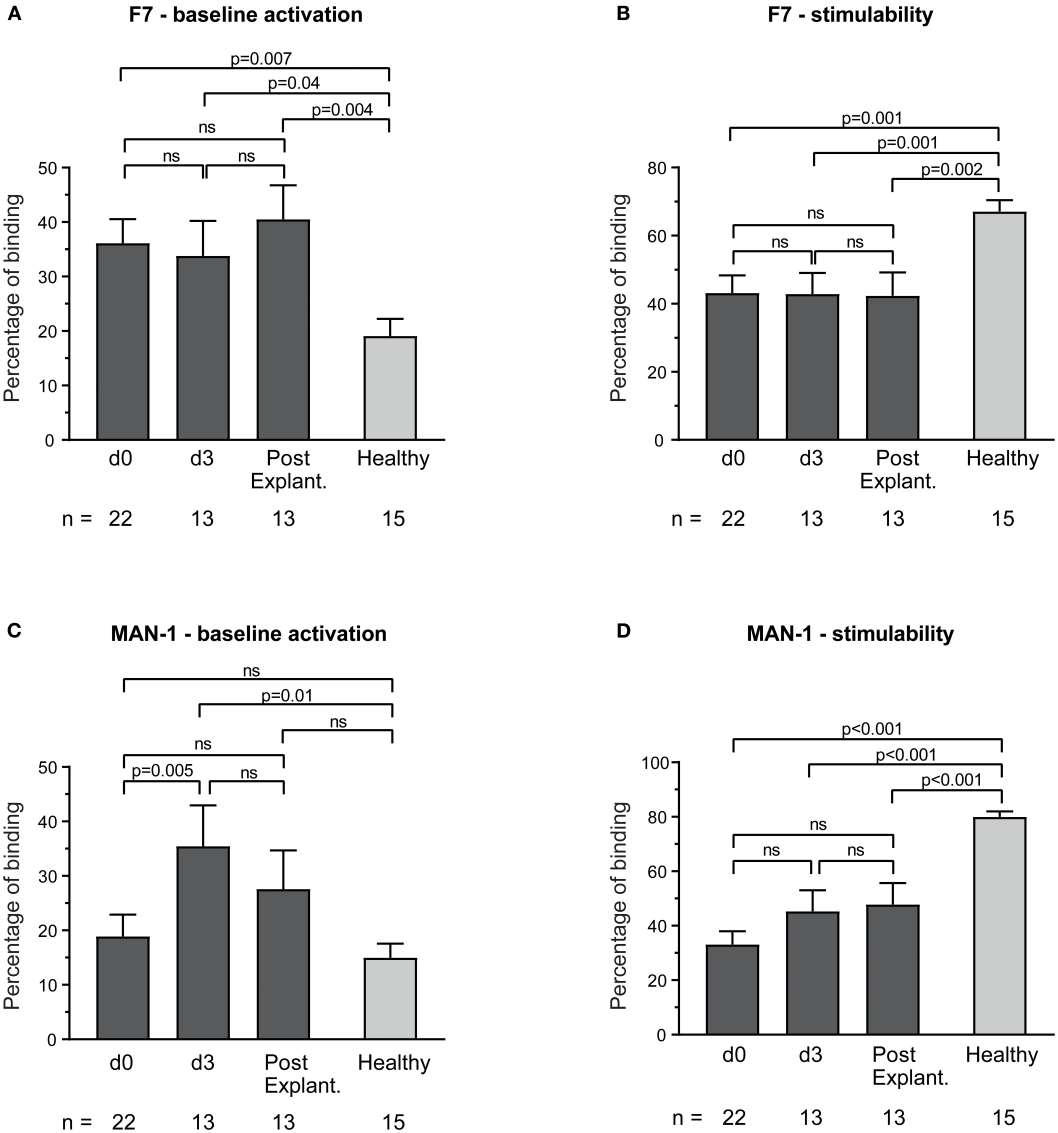
Baseline monocyte activation and stimulability as determined by F7 binding **(A,B)** and MAN-1 **(C,D)**. The number of remaining patients analyzed at each time point during VA-ECMO therapy (d0, d3) and after explantation (post explant.) is presented below the individual bars. Baseline monocyte activation was measured in phosphate buffered saline treated samples. Stimulability was assessed in samples treated with phorbol 12-myristate 13-acetate to induce maximum monocyte activation. Binding of F7 and MAN-1 to CD14^+^ monocytes is presented in percentage and was quantified as described in the Materials and Methods section. Mixed effects models were used to analyze differences between means across different time points (d0, d3, and post explantation vs. each other). Unpaired *t*-tests were used to analyze differences of means at single time points. ns, not significant. Data are presented as mean ± SEM.

The highly-activation-specific and conformationally sensitive anti-Mac-1 binding protein F7 showed increased baseline monocyte activation compared to healthy controls (e.g., percentage binding: VA-ECMO day 3 vs. healthy controls: 33.8 ± 6.4 vs. 19.1 ± 3.1, *p* = 0.04, Figure 1A). The conformationally sensitive anti-Mac-1 binding protein MAN-1 also detected increased baseline monocyte activation in VA-ECMO patients, but only on day 3 (MAN-1: 35.5 ± 7.5 vs. 15.0 ± 2.6, p=0.01, Figure 1C). Monocyte stimulability on ECMO and even after explantation using these parameters was significantly reduced compared to healthy controls (e.g., percentage binding VA-ECMO day 0 vs. healthy controls F7: 43.1 ± 5.2 vs. 67.1 ± 3.3, *p* = 0.001, MAN-1: 33.1 ± 4.1 vs. 80.0 ± 2.0, *p* < 0.001, Figures 1B,D).

**Figure 2:**
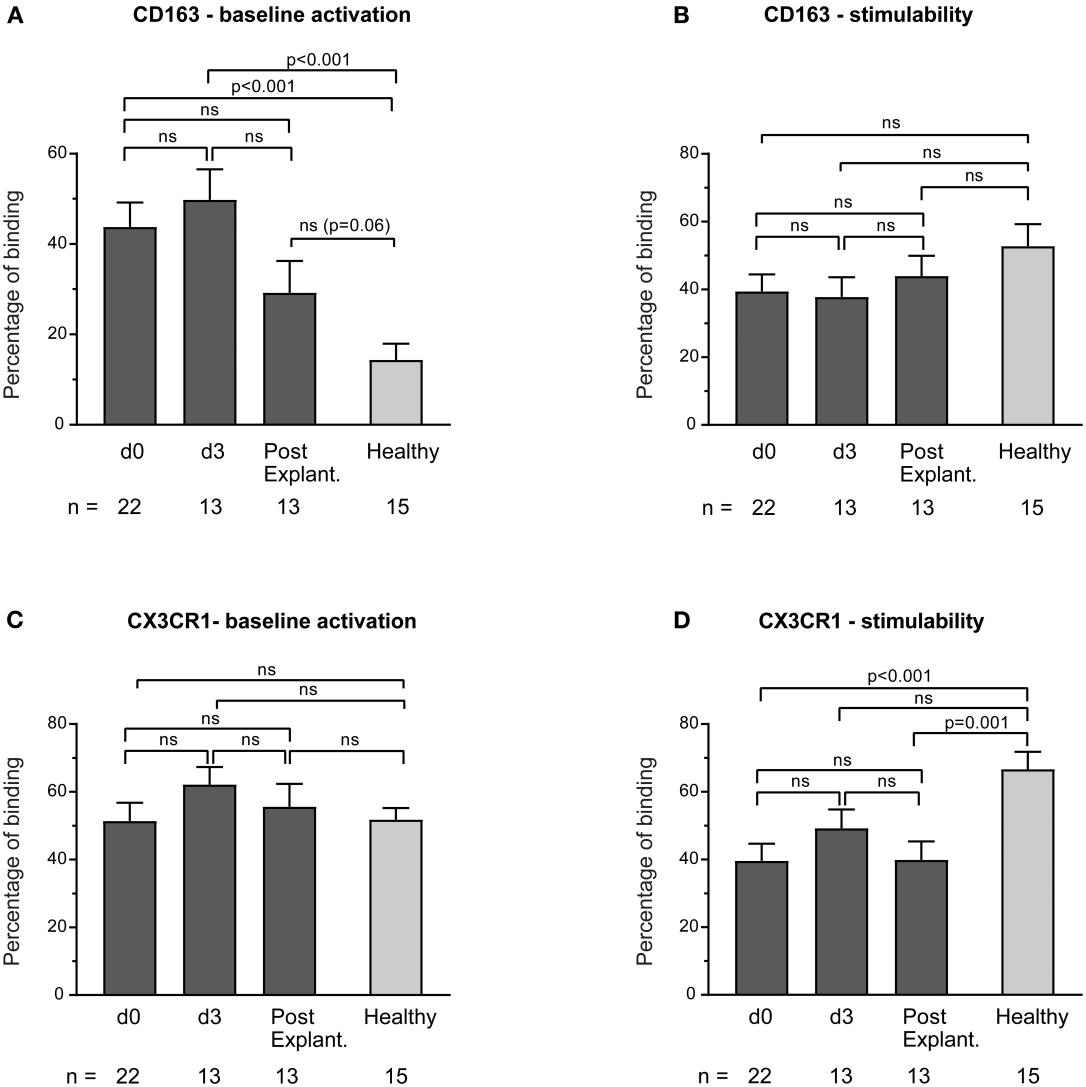
Baseline monocyte activation and stimulability as determined by CD163 **(A,B)** and CX3CR1 **(C,D)** surface expression. The number of remaining patients analyzed at each time point during VA-ECMO therapy (d0, d3) and after explantation (post explant.) is presented below the individual bars. Baseline monocyte activation was measured in phosphate buffered saline treated samples. Stimulability was assessed in samples treated with phorbol 12-myristate 13-acetate to induce maximum monocyte activation. CD14^+^ monocyte CD163 and CX3CR1 expression is presented in percentage and was quantified as described in the Materials and Methods section. Mixed effects models were used to analyze differences between means across different time points (d0, d3, and post explantation vs. each other). Unpaired *t*-tests were used to analyze differences of means at single time points. ns, not significant. Data are presented as mean ± SEM.

Baseline monocyte activation according to the expression of CD163 in VA-ECMO patients was significantly increased compared to healthy controls (e.g., percentage binding VA-ECMO day 0 vs. healthy controls: CD163: 43.8 ± 5.4 vs. 14.4 ± 3.6, *p* < 0.001), whereas stimulability was not significantly different (e.g., percentage binding VA-ECMO day 0 vs. healthy controls: CD163: 39.4 ± 5.0 vs. 52.7 ± 6.5, *p* = 0.11, not significant, Figures 2A,B). Using CX3CR1 we found similar baseline monocyte activation in monocytes compared to healthy controls (e.g., percentage binding VA-ECMO day 0 vs. healthy controls: CX3CR1: 51.4 ± 5.4 vs. 51.8 ± 3.8, *p* = 0.95, not significant), but reduced stimulability (e.g., percentage binding VA-ECMO day 0 vs. healthy controls: CX3CR1: 39.6 ± 5.1 vs. 66.7 ± 5.2, *p* < 0.001, Figures 2C,D).

**Figure 3:**
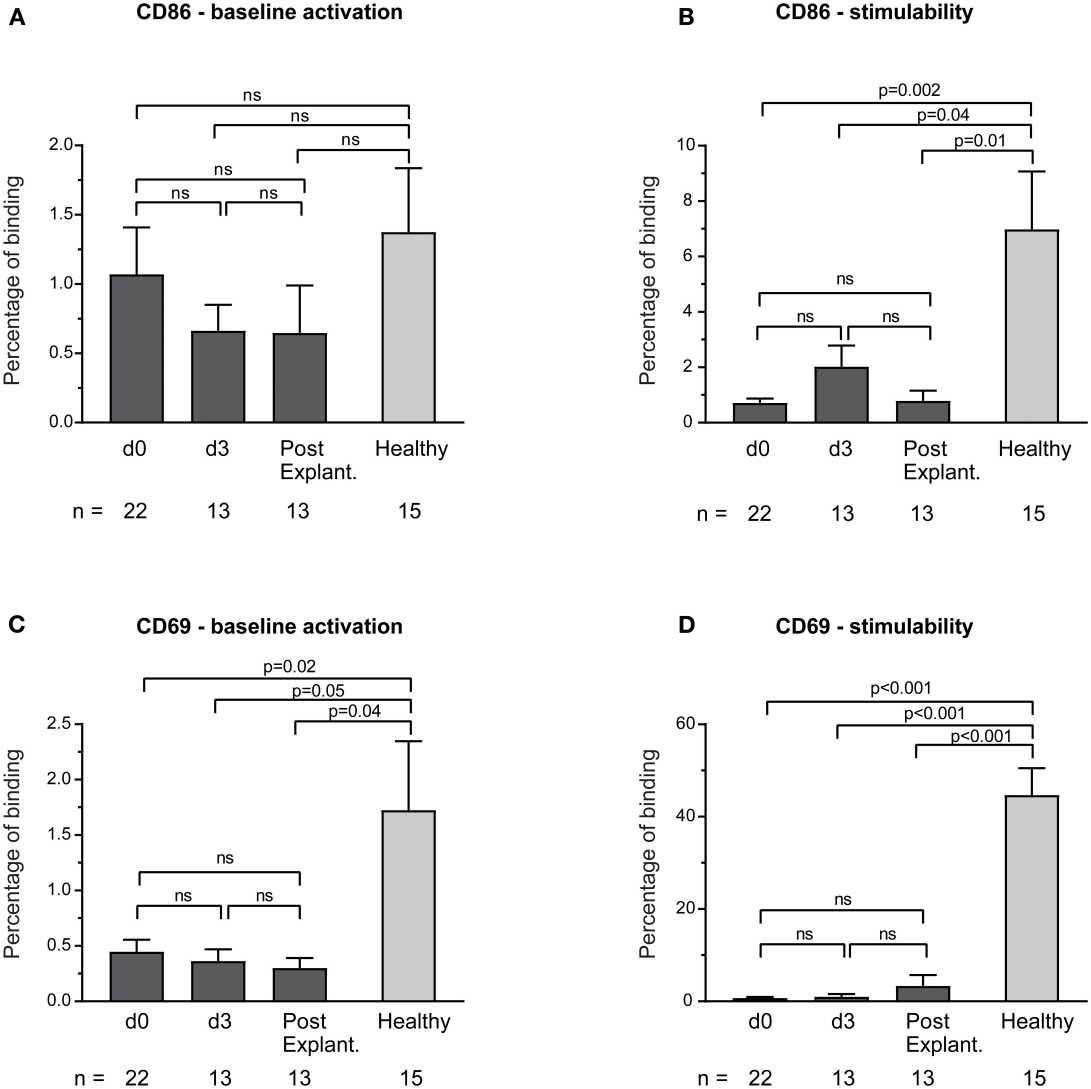
Baseline monocyte activation and stimulability as determined by CD86 **(A,B)** and CD69 **(C,D)** surface expression. The number of remaining patients analyzed at each time point during VA-ECMO therapy (d0, d3) and after explantation (post explant.) is presented below the individual bars. Baseline monocyte activation was measured in phosphate buffered saline treated samples. Stimulability was assessed in samples treated with phorbol 12-myristate 13-acetate to induce maximum monocyte activation. CD14^+^ monocyte CD69 and CD86 surface expression is presented in percentage and was quantified as described in the Materials and Methods section. ns, not significant. Baseline expression levels of CD86 and CD69 were low in healthy controls and patients. Mixed effects models were used to analyze differences between means across different time points (d0, d3, and post explantation vs. each other). Unpaired *t*-tests were used to analyze differences of means at single time points. Data are presented as mean ± SEM.

CD69 and CD86 surface expression on unstimulated monocytes in both healthy controls and VA-ECMO patients was low (Figures 3A,C). Although monocyte stimulability was preserved in healthy controls (Supplementary Figure 1), monocytes in VA-ECMO patients showed virtually no signs of stimulability (e.g., percentage binding VA-ECMO day 0 vs. healthy controls CD86: 0.7 ± 0.2 vs. 7.0 ± 2.1, *p* = 0.001, CD69: 0.7 ± 0.2 vs. 44.7 ± 5.8, *p* < 0.001, Figures 3B,D).

**Figure 4:**
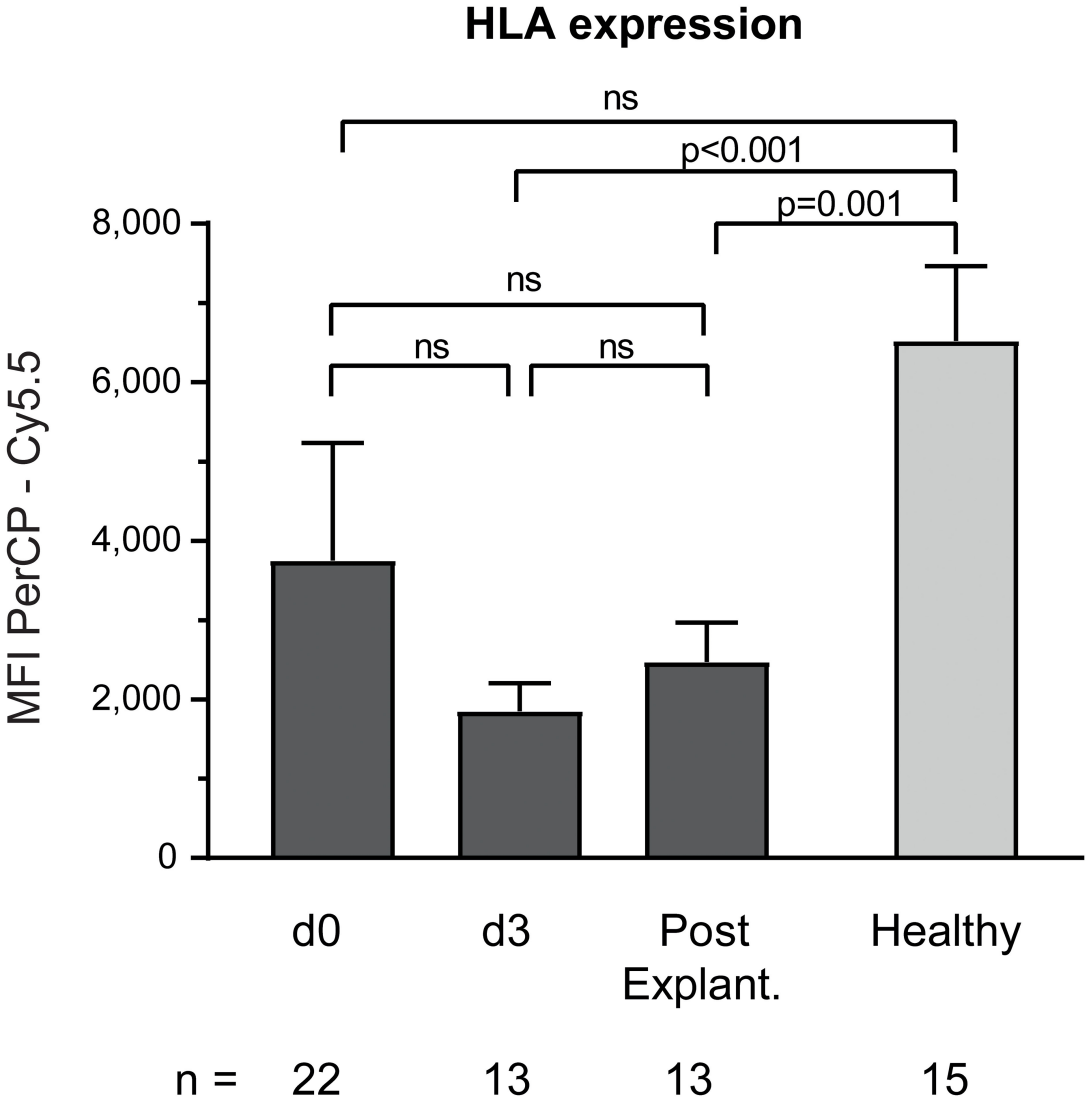
Baseline monocyte HLA expression on CD14^+^ monocytes. The number of remaining patients analyzed at each time point during VA-ECMO therapy (d0, d3) and after explantation (post explant.) is presented below the individual bars. HLA expression was quantified as the mean fluorescence intensity (MFI) in the PerCP—Cy5.5 channel and was measured in phosphate buffered saline treated samples as described in the Materials and Methods section. ns, not significant. Mixed effects models were used to analyze differences between means across different time points (d0, d3, and post explantation vs. each other). Unpaired *t*-tests were used to analyze differences of means at single time points. Data are presented as mean ± SEM.

Baseline monocyte HLA-expression was significantly decreased in VA-ECMO patients, particularly on day 3 (MFI PerCP—Cy5.5 VA-ECMO day 3 vs. healthy controls: 1,852 ± 352.3 vs. 6,523 ± 939.7, *p* < 0.001, Figure 4).

The comparison of baseline activation and stimulability of monocytes at day 0 between survivors and non-survivors is presented in Table 2. Clinical characteristics and laboratory parameters of survivors and non-survivors are presented in Supplementary Tables 1, 2. Baseline monocyte activation as determined by DARPin F7 was significantly decreased in non-survivors (*p* = 0.03). The area under the ROC curve for mortality was 0.802 (*p* = 0.02). Moreover, non-survivors also showed a clear trend toward reduced monocyte stimulability as determined by F7 (*p* = 0.06). The area under the ROC curve for mortality was 0.752 (*p* = 0.05). Other parameters, including MAN-1, did not show relevant differences in baseline monocyte activation or stimulability between survivors and non-survivors.

**Table 2:** Parameters of monocyte function and distribution in survivors vs. non-survivors on day 0.

**Parameter**	**Survivor**	**Non-survivor**	***P*-value**
F7 (–) [%]	45.4 ± 5.7	26.9 ± 5.7	**0.03**
MAN-1 (–) [%]	22.0 ± 7.6	15.8 ± 2.7	0.45
CD163 (–) [%]	45.1 ± 7.7	42.5 ± 8.0	0.82
CX3CR1 (–) [%]	50.7 ± 8.4	52.1 ± 7.2	0.91
CD69 (–) [%]	0.3 ± 0.1	0.6 ± 0.2	0.06
CD86 (–) [%]	0.4 ± 0.1	1.1 ± 0.6	0.19
HLA (–) [MFI]	2058 ± 317	2289 ± 371	0.64
F7 (+) [%]	52.8 ± 7.6	33.4 ± 6.1	0.06
MAN-1 (+) [%]	39.3 ± 7.4	26.9 ± 6.1	0.21
CD163 (+) [%]	43.3 ± 7.8	35.5 ± 6.5	0.45
CX3CR1 (+) [%]	37.9 ± 7.5	41.3 ± 7.1	0.75
CD69 (+) [%]	1.0 ± 0.4	0.4 ± 0.1	0.20
CD86 (+) [%]	0.9 ± 0.3	0.9 ± 0.3	0.98
Total monocytes/ml	231,152 ± 42,618	352,547 ± 103,541	0.29
Classical monocytes/ml	151,932 ± 30,503	281,890 ± 85,492	0.15
Intermediate monocytes/ml	27,345 ± 6,394	119,940 ± 45,829	**0.05**
Non-classical monocytes/ml	13,761 ± 3,994	18,971 ± 5,620	0.45
Classical monocytes [%]	65.6 ± 3.9	56.2 ± 5.8	0.19
Intermediate monocytes [%]	14.7 ± 2.8	20.6 ± 3.7	0.22
Non-classical Monocytes [%]	5.9 ± 1.2	4.9 ± 0.9	0.47

*Baseline monocyte activation is indicated by (–), monocyte stimulability is indicated by (+). Survivors were defined as patients weaned off VA-ECMO and discharged from the intensive care ward. Monocyte activation was defined as described in the Materials and Methods section. p-values were calculated by an unpaired t-test. Significant p-values are written in bold. Data are presented as mean ± SEM. HLA, human leukocyte antigen; MFI, mean fluorescence intensity*.

Markers of monocyte function on day 0 were also compared between patients receiving VA-ECMO due to cardiogenic shock and those receiving VA-ECMO due to eCPR (Supplementary Table 3). Clinical characteristics of these two groups are presented in Supplementary Tables 4, 5. No significant differences regarding monocyte function were found between these groups.

**Figure 5:**
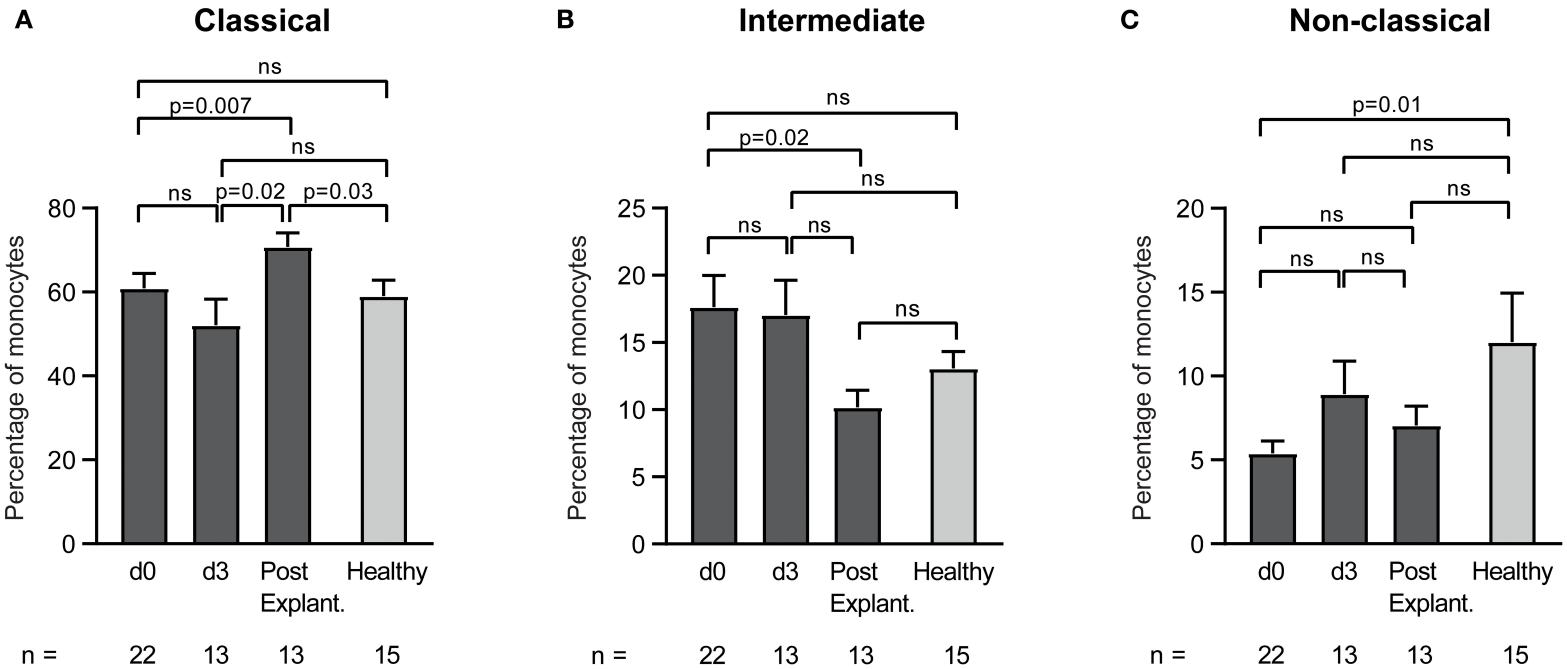
Distribution of monocyte subsets in patients receiving veno-arterial extracorporeal membrane oxygenation on different days (d0, d3) and after explantation (post explant.) compared to healthy controls. The percentage of classical **(A)**, intermediate **(B)**, and non-classical **(C)** monocytes is presented. The number of remaining patients at every time point is indicated below. Data are presented as mean ± SEM. Mixed effects models were used to analyze differences between means across different time points (d0, d3, and post explantation vs. each other). Unpaired *t*-tests were used to analyze differences of means at single time points.

Furthermore, monocyte heterogeneity in VA-ECMO patients according to the surface expression of CD14 and CD16 was investigated. Classical (CD14^++^CD16^−^), intermediate (CD14^++^CD16^+^), and non-classical monocytes (CD14^+^CD16^++^) were differentiated (Figure 5). We found shifts in the percentages of the three monocyte subsets during different days on ECMO and after weaning off ECMO. While there was a significant increase of classical monocytes from day 0 until after explantation (percentage of classical monocytes: day 0 vs. after explantation: 60.9 ± 3.5 vs. 70.7 ± 3.3, *p* = 0.007), intermediate monocytes decreased during this time (percentage of intermediate monocytes day 0 vs. after explantation: 17.6 ± 2.4 vs. 10.1 ± 1.3, *p* = 0.02). The percentage of non-classical monocytes in VA-ECMO patients did not significantly change at any time point. Compared to healthy controls, several differences in the distribution of monocyte populations were observed. For example, the percentage of classical monocytes was significantly increased after explantation (percentage of classical monocytes after explantation vs. healthy controls: 70.7 ± 3.3 vs. 59.0 ± 3.8, *p* = 0.03) and non-classical monocytes were significantly decreased on day 0 compared to healthy controls (percentage of non-classical monocytes day 0 vs. healthy controls: 5.4 ± 0.7 vs. 12.0 ± 2.9, *p* = 0.01).

**Figure 6:**
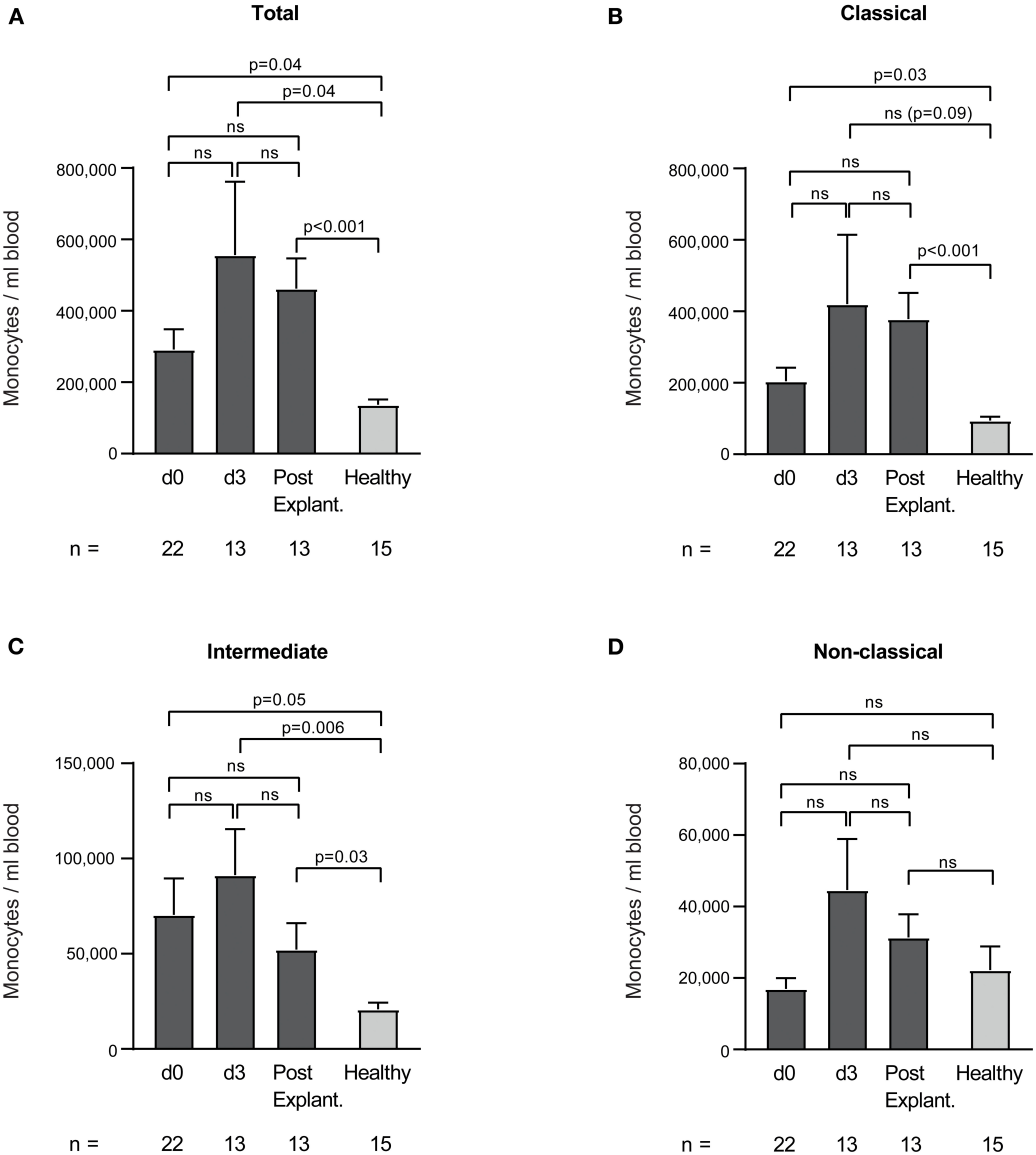
Total **(A)**, classical **(B)**, intermediate **(C)**, and non-classical **(D)** monocyte counts per ml blood in patients receiving veno-arterial extracorporeal membrane oxygenation on different days (d0, d3) and after explantation (post explant.) compared to healthy controls. The number of remaining patients at every time point is indicated below. Data are presented as mean ± SEM. Mixed effects models were used to analyze differences between means across different time points (d0, d3, and post explantation vs. each other). Unpaired *t*-tests were used to analyze differences of means at single time points.

Absolute monocyte counts of VA-ECMO patients did not significantly change during ECMO and after explantation. As expected, however, we found large differences compared to healthy controls (Figure 6). Total monocyte count on all days was significantly increased compared to healthy controls (e.g., total monocyte count/ml day 0 vs. healthy controls: 291,850 ± 56,218 vs. 137,180 ± 14,797, *p* = 0.04). Classical (except day 3) and intermediate monocytes counts were also significantly increased (monocyte count/ml day 0 vs. healthy controls: classical: 204,523 ± 37,798 vs. 94,126 ± 11,148, *p* = 0.03, intermediate: 70,386 ± 19,172 vs. 20,851 ± 3,482). Non-classical monocyte counts did not differ significantly from healthy monocytes.

Interestingly, we found significantly increased intermediate monocyte counts in non-survivors compared to survivors (Table 2). However, the area under the ROC curve for mortality was only 0.587 (not significant).

## Discussion

Using several markers of monocyte function, our data show increased baseline monocyte activation and reduced monocyte stimulability in patients receiving VA-ECMO suggesting monocytes are *dysfunctional*. Amongst the different markers used to identify monocyte dysfunction, the *unique tool* DARPin F7 is the most promising as low levels of binding on day 0 were predictive of mortality.

A unifying characteristic of monocyte dysfunction in VA-ECMO patients was the reduced stimulability of monocytes which was observed for all markers except CD163. It is possible, that with a larger sample size a significant difference in stimulability could have also been observed for CD163. In contrast, increased baseline monocyte activation in VA-ECMO patients was detected using our *unique* activation-specific anti-Mac-1 binding proteins F7 and MAN-1 and the surface marker CD163. These findings need not be contradictory as these markers report different aspects of monocyte function that may differ and findings supporting both activation and immunocompromise have been previously described in a group of critically-ill septic patients (24).

Monocyte dysfunction as demonstrated in this study may be associated with a general *immunoparalysed state* in VA-ECMO patients. *Immunoparalysis* was recently reported in a heterogeneous group of adult and neonatal VV- and VA-ECMO patients based on findings of cytokine levels from LPS-stimulated whole blood (4).

Immunoparalysis in VA-ECMO patients is supported by several of our findings, such as decreased CX3CR1 expression in response to stimulation and reduced HLA-expression. Decreased expression of CX3CR1 was previously reported in severely septic patients and regarded as evidence of immunosuppression (25) while reduced monocyte HLA expression is often associated with immunoparalysis in critical illness, for example after cardiopulmonary bypass (26, 27). Moreover, our results show there was virtually a loss of CD86 and CD69 expression in response to stimulation in VA-ECMO patients possibly also reflecting immunoparalysis. While there is little information on monocyte CD69 expression in critically ill patients, a recent study found reduced monocyte CD86 stimulability to be associated with immunoparalysis in patients after cardiopulmonary bypass (28).

How the phenomenon of immunoparalysis develops in VA-ECMO patients is not clear, but most likely it is caused by a combination of factors, e.g., the large extracorporeal surfaces triggering immune cell activation and the severe underlying illness leading to ECMO therapy (29).

Importantly, immunoparalysis has been claimed to be associated with worse outcomes in critically ill patients, particularly in sepsis (30, 31) and recently, Combes et al. claimed that it might also be associated with worse outcomes in ECMO patients (32).

Our data is in line with these findings as monocyte dysfunction determined by the *unique tool* F7 was related to mortality. Baseline binding of F7 to monocytes from VA-ECMO patients on day 0 was significantly lower in non-survivors indicating more severe monocyte dysfunction. Logistic regression and analysis of area under the ROC curve demonstrated that low binding of F7 on day 0 was predictive of increased mortality. To the best of our knowledge, we are the first group to demonstrate this link between monocyte dysfunction in VA-ECMO patients and mortality. Therefore, in the future, monocyte dysfunction detected by F7 may be used as a novel *biomarker* guiding early clinical decision making.

Our results are supported by a previous study on septic patients in which low monocytic Mac-1 expression, as determined by a conventional anti-Mac-1 IgG antibody, was associated with poor outcome and an anti-inflammatory response syndrome which is commonly seen as an early stage of immunoparalysis (33). Analyzing monocytic Mac-1 expression and conformation is therefore well-suited to detect monocyte activation, -dysfunction and immunoparalysis in critically ill patients as it translates into clinical outcome.

Although F7 and MAN-1 both bind to the activated conformation of Mac-1, they do not share the same epitope and could be detecting slightly different conformational states of Mac-1. This could explain why increased baseline monocyte activation was detected at all time points by F7, but only on day 3 by MAN-1. In clinical practice, DARPin F7 may be advantageous compared to conventional anti-Mac-1 antibodies and even MAN-1 due to its inherently high sensitivity and stability, low cost, and ease of production (34).

To the best of our knowledge, this is the first study to investigate the distribution of monocyte subsets in VA-ECMO patients. We report significant changes in the proportion of classical and intermediate monocytes in patients on VA-ECMO compared to after ECMO explantation. In addition, we show increased total, classical, and intermediate absolute monocyte counts in patients receiving VA-ECMO and after explantation compared to healthy controls. Classical and intermediate monocytes are now widely recognized as pro-inflammatory mediators (35) and proportional changes could reflect the inflammatory reaction to VA-ECMO.

As we focused our study on monocytes, we cannot exclude that other leukocyte subsets were also increased. This is possible, given the inflammatory reaction in these patients but the median total white blood cell count on day 0 determined using automated cell counting in our central laboratory was within the upper normal range. This is in line with previous reports of lymphopenia, reduced or only stable neutrophil counts in ECMO patients (36) and emphasizes the finding of increased absolute monocyte counts in our study which could reflect the importance of monocytes for the inflammatory reaction and outcome in VA-ECMO patients. In this context, intermediate monocytes were significantly increased in non-survivors indicating a possible detrimental role of this monocyte subset. Our data is in line with a study reporting increased levels of intermediate monocytes in patients with poor outcome after cardiac surgery (37). Further studies, however, are required to characterize the functional role of the different monocyte subsets in VA-ECMO patients in detail.

This study is not without limitations. Since it did not include a pre-ECMO time point the direct effect of ECMO initiation on monocyte function cannot be clearly determined. As this was an exploratory study, we used healthy, unmatched controls as a control group and therefore some of the results may have been influenced by the underlying disease. For example, since previous studies of septic shock and post cardiopulmonary bypass found monocyte dysfunction to be associated with immunocompromise, monocyte dysfunction in this study may be indicative of shock severity rather than use of VA-ECMO itself. Future studies would benefit from larger sample sizes and more suitable controls, such as patients with cardiogenic shock without ECMO support. Since all patients received transfusions in this study, we cannot exclude results were affected. Moreover, as this study only included a relatively small number of ECMO patients, it cannot be excluded that different ECMO circuits affected results.

## Conclusion

Monocytes from VA-ECMO patients are dysfunctional as baseline monocyte activation is significantly increased but monocyte stimulability is decreased. Distribution of monocyte subsets changes in patients receiving VA-ECMO over time and absolute classical and intermediate monocyte counts are significantly elevated compared to controls. Monocyte dysfunction, as determined by the *unique tool* DARPin F7 could be valuable for predicting mortality in patients receiving VA-ECMO and may be used as a novel biomarker guiding early clinical decision making in the future. Monocyte dysfunction, as demonstrated in this study for VA-ECMO patients, has been previously associated with immunocompromise in patients with septic and post-cardiotomy shock. Clinicians may therefore evaluate a lower threshold to initiate antimicrobial therapy in patients with cardiogenic shock severe enough to require VA-ECMO.

## Data Availability Statement

The raw data supporting the conclusions of this article will be made available by the authors upon reasonable request.

## Ethics Statement

The studies involving human participants were reviewed and approved by Ethics Committee of the University of Freiburg. The patients or their legal representatives provided their written informed consent to participate in this study.

## Author Contributions

PS: study design, acquisition, analysis and interpretation of data, preparation of manuscript. LO: acquisition and analysis of data. IB and MM: analysis and interpretation of data. KK: statistical counseling, analysis of data, and preparation of manuscript. TW and JE: data analysis, interpretation and preparation of manuscript. GT and CB: study design and interpretation of data. KP: study design, analysis and interpretation of data. PD: study design, analysis and interpretation of data, preparation of manuscript. All authors proof-read and accepted the final draft of the manuscript.

## Conflict of Interest

The authors declare that the research was conducted in the absence of any commercial or financial relationships that could be construed as a potential conflict of interest.
