# Supplementary material for: Monocyte Dysfunction Detected by the Designed Ankyrin Repeat Protein F7 Predicts Mortality in Patients Receiving Veno-Arterial Extracorporeal Membrane Oxygenation
Source: Front Cardiovasc Med. 2021 Jul 19;8:689218. doi: 10.3389/fcvm.2021.689218 (PMC8326337; doi:10.3389/fcvm.2021.689218)
Supplement: Supplementary file 1 [file Data_Sheet_1.PDF]

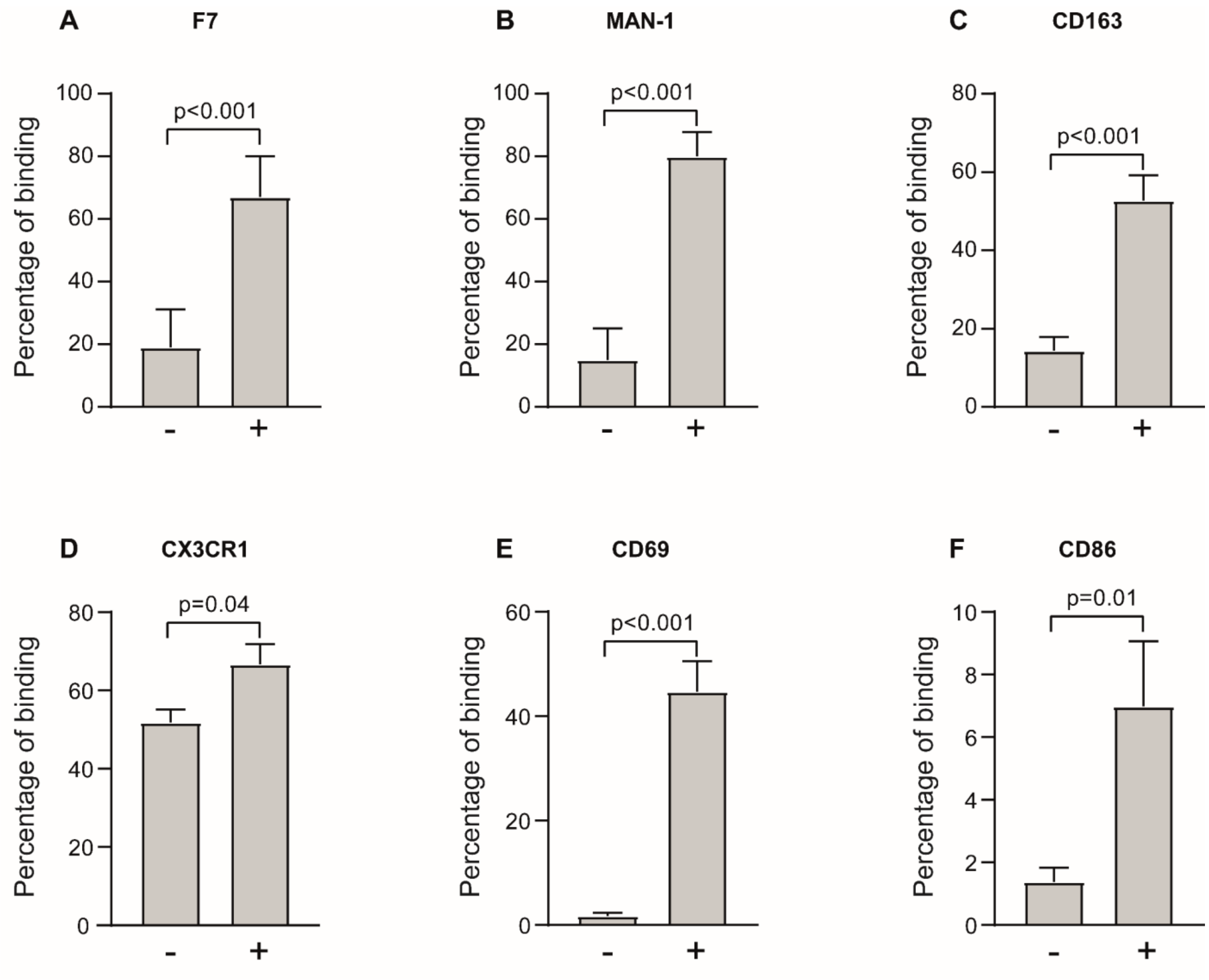


**Supplementary Figure S1.** Assessing the capacity of different antibodies and binding proteins to detect monocyte activation in healthy volunteers (**A-F**). Baseline monocyte activation was measured in samples treated with phosphate buffered saline (-). Activated monocytes (+) were stimulated with phorbol 12-myristate 13-acetate to induce activation. Increased activation-dependent binding or expression could be confirmed in healthy controls. Binding is presented as percentage and was quantified by flow cytometry as described in the Materials and Methods section. n=15, p-values were calculated by unpaired Student’s tests. Data are presented as mean±SEM.
