# Supplementary material for: Monocyte Dysfunction Detected by the Designed Ankyrin Repeat Protein F7 Predicts Mortality in Patients Receiving Veno-Arterial Extracorporeal Membrane Oxygenation
Source: Front Cardiovasc Med. 2021 Jul 19;8:689218. doi: 10.3389/fcvm.2021.689218 (PMC8326337; doi:10.3389/fcvm.2021.689218)
Supplement: Supplementary file 2 [file Data_Sheet_2.PDF]

Parameter	Survivors	Non-survivors	p-value
Patients, n (%)	11 (50)	11 (50)	n/a
Age, y (Q1-Q3)	66 (52-72)	59 (52-75)	0.92
Female, n (%)	4 (36)	4 (36)	>0.99
Type of VA-ECMO, n (%)			
Stöckert Sorin	7 (64)	5 (45)	0.67
Maquet	2 (18)	5 (45)	0.36
Deltastream	1 (9)	1 (9)	>0.99
CARL	1 (9)	0 (0)	>0.99
Days on ECMO (median, Q1-Q3)	7 (4-7)	4 (3-8)	0.61
ECMO Blood Flow (l/min, Q1-Q3)	4.5 (3.6-5.0)	4.3 (4.1-4.7)	0.62
Indication for VA-ECMO, n (%)			
Cardiogenic shock	8 (73)	7 (64)	>0.99
eCPR	3 (27)	4 (36)	>0.99
Coronary heart disease, n (%)	8 (73)	7 (64)	>0.99
Atrial fibrillation, n (%)	5 (45)	3 (27)	0.66
Diabetes mellitus, n (%)	2 (18)	2 (18)	>0.99
Hypertension, n (%)	4 (36)	3 (27)	>0.99
Active smoker, n (%)	2 (18)	1 (9)	>0.99
Hypercholesterolemia, n (%)	3 (27)	1 (9)	0.59
Cancer, n (%)	0 (0)	0 (0)	>0.99
Acute renal failure, n (%)	4 (36)	11 (100)	**0.004**
Continuous hemodialysis, n (%)	2 (18)	7 (64)	0.08
Heparin, n (%)	11 (100)	10 (91)	>0.99
Dual anti-platelet therapy, n (%)	3 (27)	6 (55)	0.39
Immunosuppression (steroids), n(%)	0 (0)	1 (9)	>0.99
Received transfusions, n (%)	11 (100)	11 (100)	>0.99
Cytosorb, n (%)	0 (0)	1 (9)	>0.99
Mechanical ventilation, n (%)	11 (100)	11 (100)	>0.99
SOFA score	11.0 (9.0-11.0)	11.0 (10.0-15.0)	0.26

Supplementary Table S1. Clinical characteristics of VA-ECMO patients: survivors vs. non-survivors day 0. Data are presented as median (interquartile range) or number of patients (%). Denominator of the percentage is the total number of subjects in the group. Parameters from the patient data management system that were closest to the time point of blood sampling for flow cytometric analysis are presented. p-values were calculated by an unpaired Student's t-test for continuous data or Fisher's exact test for categorical data. Significant p-values are highlighted in bold. eCPR=extracorporeal cardiopulmonary resuscitation, SOFA=sequential organ failure assessment score.

**Parameter**	**Survivors**	**Non-survivors**	**p-value**
WBC (x10^3 /µl, Q1-Q3)	8.8 (5.3-10.4)	10.8 (8.5-21.1)	**0.02**
Platelets (x10^3 /µl, Q1-Q3)	113.0 (82.0-155.0)	97.0 (61.0-135.0)	0.36
Hb (g/dl, Q1-Q3)	8.7 (8.3-8.9)	8.6 (8.3-9.4)	>0.99
Creatinine (mg/dl, Q1-Q3)	1.0 (0.9-2.2)	2.3 (1.0-2.7)	0.23
Urea (mg/dl, Q1-Q3)	39.0 (24.0-77.0)	61.5 (50.3-90.8)	0.18
Bilirubin (mg/dl, Q1-Q3)	2.6 (1.3-2.8)	2.3 (1.5-3.6)	0.31
AST (U/l, Q1-Q3)	142.0 (36.0-225.0)	390.0 (87.0-870.0)	0.22
ALT (U/l, Q1-Q3)	58.0 (18.0-102.0)	81.0 (28.0-264.0)	0.20
CRP (mg/l, Q1-Q3)	49.0 (25.1-72.2)	70.6 (24.1-103.1)	0.67
IL-6 (pg/ml)	356.0 (272.5-725.0)	494.0 (309.0-5043)	0.15
Ferritin (ng/ml, Q1-Q3)	289.0 (173.0-924.5)	1583 (256.0-10203)	0.26
Lactate (mmol/l, Q1-Q3)	2.4 (1.3-4.2)	4.7 (1.4-10.0)	**0.02**
p_aO_2 (mmHg, Q1-Q3)	104.0 (78.0-129.0)	127.0 (73.0-221.0)	0.20
p_aCO_2 (mmHg, Q1-Q3)	39.9 (38.0-46.8)	37.2 (34.5-45.4)	0.52
F_iO (%, Q1-Q3)	50.0 (40.0-50.0)	50.0 (40.0-50.0)	0.37
PEEP (mbar, Q1-Q3)	8.0 (7.0-8.0)	9.0 (8.0-10.0)	0.25
Respiratory rate (/min, Q1-Q3)	14.5 (11.3-17.5)	14.0 (12.0-19.0)	0.42

Supplementary Table S2. Laboratory parameters and ventilation settings from patients on VA-ECMO patients: survivors vs. non-survivors day 0. Data are presented as median (interquartile range). Denominator of the percentage is the total number of subjects in the group. Parameters from the patient data management system that were closest to the time point of blood sampling for flow cytometric analysis are presented. p-values were calculated by an unpaired Student’s t-test. Significant p-values are highlighted in bold. ALT=alanine aminotransferase, AST=aspartate aminotransferase, CRP=C-reactive protein, F_iO=fraction of inspired oxygen, PEEP=positive endexpiratory pressure, WBC=white blood cells.

Parameter	Cardiogenic Shock	eCPR	p-value
F7 (-) [%]	34.3 ± 5.6	40.1 ± 7.2	0.55
MAN-1 (-) [%]	19.5 ± 5.8	17.6 ± 3.0	0.84
CD163 (-) [%]	39.6 ± 7.1	52.6 ± 7.3	0.27
CX3CR1 (-) [%]	48.9 ± 7.3	56.7 ± 6.7	0.51
CD69 (-) [%]	0.4 ± 0.1	0.6 ± 0.3	0.28
CD86 (-) [%]	0.8 ± 0.3	1.6 ± 0.8	0.25
HLA (-) [MFI]	2,102 ± 260.2	7,283 ± 4,566	0.11
F7 (+) [%]	37.0 ± 6.0	56.1 ± 8.5	0.09
MAN-1 (+) [%]	32.8 ± 6.4	33.8 ± 7.6	0.93
CD163 (+) [%]	36.2 ± 6.5	46.3 ± 7.4	0.36
CX3CR1 (+) [%]	35.1 ± 6.0	49.3 ± 8.9	0.20
CD69 (+) [%]	0.6 ± 0.2	1.0 ± 0.6	0.36
CD86 (+) [%]	0.6 ± 0.2	1.0 ± 0.4	0.31
Total monocytes /ml	333,238 ± 73,844	203,161 ± 74,685	0.29
Classical monocytes /ml	234,766 ± 48,386	139,718 ± 54,619	0.25
Intermediate monocytes /ml	82,551 ± 26,341	44,318 ± 20,203	0.37
Non-classical Monocytes /ml	15,921 ± 3,356	19,124 ± 6,730	0.64
Classical monocytes [%]	65.5 ± 2.1	51.2 ± 9.6	0.06
Intermediate monocytes [%]	18.5 ± 2.8	15.7 ± 4.5	0.59
Non-classical Monocytes [%]	5.2 ± 0.8	5.8 ± 1.5	0.74

Supplementary Table S3. Parameters of monocyte function and distribution in patients receiving VA-ECMO for cardiogenic shock vs. eCPR on day 0. Baseline monocyte activation is indicated by (-), monocyte stimulability is indicated by (+). Monocyte activation was defined as described in the Materials and Methods section. p-values were calculated by an unpaired t-test. Data are presented as mean±SEM. HLA – human leukocyte antigen, MFI – mean fluorescence intensity.

Parameter	Cardiogenic Shock	eCPR	p-value
Patients, n (%)	15 (100)	7 (100)	-
Age, y (Q1-Q3)	64 (52-75)	59 (52-66)	0.68
Survivors	8 (53)	3 (43)	0.99
Female, n (%)	5 (33)	3 (43)	0.99
Type of VA-ECMO, n (%)<br>Stöckert Sorin<br>Maquet<br>Deltastream<br>CARL	 8 (53)<br>4 (27)<br>2 (13)<br>1 (7)	 4 (57)<br>3 (43)<br>0 (0)<br>0 (0)	 >0.99<br>0.63<br>0.99<br>>0.99
Days on ECMO (median, Q1-Q3)	6 (4-7)	5 (3-8)	0.58
ECMO Blood Flow (l/min, Q1-Q3)	4.4 (3.4-5.0)	4.3 (4.1-4.5)	0.99
Cardiovascular disease, n (%)	9 (60)	6 (86)	0.35
Atrial fibrillation, n (%)	6 (40)	2 (29)	0.99
Diabetes mellitus, n (%)	1 (7)	1 (14)	0.99
Hypertension, n (%)	6 (40)	1 (14)	0.35
Active smoker, n (%)	2 (13)	1 (14)	0.99
Hypercholesterolemia, n (%)	3 (20)	1 (14)	0.99
Cancer, n (%)	0 (0)	0 (0)	0.99
Acute renal failure, n (%)	11 (73)	4 (57)	0.63
Continuous hemodialysis, n (%)	7 (47)	2 (29)	0.65
Heparin, n (%)	14 (93)	7 (100)	0.99
Dual anti-platelet therapy, n (%)	4 (27)	5 (71)	0.07
Immunosuppression (steroids), n(%)	1 (7)	0 (0)	0.99
Received transfusions, n (%)	15 (100)	7 (100)	0.99
Mechanical ventilation, n (%)	15 (100)	7 (100)	0.99
SOFA score	10.5 (8.3-11.0)	12.0 (11.0-14.0)	0.13

Supplementary Table S4. Clinical characteristics of patients receiving VA-ECMO for cardiogenic shock vs. eCPR day 0. Data are presented as median (interquartile range) or number of patients (%). Denominator of the percentage is the total number of subjects in the group. Parameters from the patient data management system that were closest to the time point of blood sampling for flow cytometric analysis are presented. p-values were calculated by an unpaired Student's t-test for continuous data or Fisher's exact test for categorical data. eCPR=extracorporeal cardiopulmonary resuscitation, SOFA=sequential organ failure assessment score.

**Parameter**	**Cardiogenic Shock**	**eCPR**	**p-value**
WBC (x10^3 /µl, Q1-Q3)	10.0 (7.7-14.3)	8.9 (4.4-10.8)	0.65
Platelets (x10^3 /µl, Q1-Q3)	113.0 (68.0-153.0)	97.0 (75.0-135.0)	0.89
Hb (g/dl, Q1-Q3)	8.6 (8.3-9.2)	8.7 (8.3-9.7)	0.71
Creatinine (mg/dl, Q1-Q3)	1.9 (0.9-2.9)	1.2 (1.0-2.5)	0.44
Urea (mg/dl, Q1-Q3)	61.5 (34.3-94.3)	51.0 (24.0-64.0)	0.30
Bilirubin (mg/dl, Q1-Q3)	2.6 (1.7-3.5)	1.5 (1.3-2.8)	0.35
AST (U/l, Q1-Q3)	119.0 (58.0-417.0)	273.0 (150.0-870.0)	0.77
ALT (U/l, Q1-Q3)	55.0 (18.0-102.0)	80.0 (36.0-264.0)	0.84
CRP (mg/l, Q1-Q3)	59.0 (29.7-104.4)	28.8 (24.1-98.8)	0.37
IL-6 (pg/ml)	351.0 (220.8-546.8)	1,126 (494-50,000)	**0.04**
Ferritin (ng/ml, Q1-Q3)	289.0 (187.0-1,717)	4,163 (287-7,571)	0.77
Lactate (mmol/l, Q1-Q3)	2.4 (1.3-4.2)	4.8 (2.9-10.0)	0.06
p_aO_2 (mmHg, Q1-Q3)	121.0 (83.0-221.0)	78.0 (69.4-138.0)	0.19
p_aCO_2 (mmHg, Q1-Q3)	38.8 (35.5-45.4)	39.9 (34.8-45.7)	0.98
F_iO (%, Q1-Q3)	40.0 (40.0-50.0)	50.0 (50.0-50.0)	**0.03**
PEEP (mbar, Q1-Q3)	8.0 (7.0-9.0)	9.0 (8.0-10.0)	0.29
Respiratory rate (/min, Q1-Q3)	14.5 (10.5-18.0)	14.0 (13.0-18.0)	0.59

Supplementary Table S5. Laboratory parameters and ventilation settings from patients receiving VA-ECMO for cardiogenic shock vs. eCPR day 0. Data are presented as median (interquartile range). Denominator of the percentage is the total number of subjects in the group. Parameters from the patient data management system that were closest to the time point of blood sampling for flow cytometric analysis are presented. p-values were calculated by an unpaired Student’s t-test. Significant p-values are highlighted in bold. ALT=alanine aminotransferase, AST=aspartate aminotransferase, CRP=C-reactive protein, F_iO=fraction of inspired oxygen, PEEP=positive endexpiratory pressure, WBC=white blood cells.
